# Interaction between the Kansas City Cardiomyopathy Questionnaire and the Pocock’s clinical score in predicting heart failure outcomes

**DOI:** 10.1007/s11136-015-1154-9

**Published:** 2015-10-11

**Authors:** Kiswendsida Sawadogo, Jérôme Ambroise, Steven Vercauteren, Marc Castadot, Michel Vanhalewyn, Jacques Col, Annie Robert

**Affiliations:** The Pôle de recherche Epidémiologie et Biostatistique, Institut de Recherche Expérimentale et Clinique (IREC-EPID), Université catholique de Louvain, Clos Chapelle-aux-Champs 30, Box B1.30.13, 1200 Brussels, Belgium; The Brussels Heart Centre (BHC), Clinique Saint Jean, Boulevard du Jardin Botanique 32, 1000 Brussels, Belgium; Société Scientifique de Médecine Générale (SSMG), Rue de Suisse 8, 1060 Brussels, Belgium

**Keywords:** Heart failure, Kansas City Cardiomyopathy Questionnaire, Quality of life

## Abstract

**Purpose:**

Heart failure (HF) is a complex syndrome. Its appropriate management should combine several health measurements. We assessed the relationship between the Kansas City Cardiomyopathy Questionnaire (KCCQ) and the Pocock’s clinical score.

**Methods:**

We conducted a prospective registry of HF outpatients. The main outcome was occurrence of death or hospitalization during a 6-month follow-up. A multivariate logistic regression was performed, including the KCCQ overall summary score, the Pocock’s clinical score and their interaction in the model.

**Results:**

From January 2008 to December 2010, 143 patients were involved. Mean age of patients was 68 years, and 74 % were men. KCCQ’s overall summary score and Pocock’s clinical score were inversely correlated (*r* = −0.24, *p* = 0.026). A total of 61 (42.7 %) events occurred. There was a high proportion of events (77.8 %) in patients with a Pocock’s clinical score >50 %, whatever the KCCQ score value. When the KCCQ score was ≤50 %, there was a low increase in risk as the Pocock’s clinical score increased (OR 2.0 [0.6; 6.6]). However, when the KCCQ score was between 50 and 75 or ≥75 %, there was a high increase in risk as the Pocock’s clinical score increased (OR 6.9 [1.2; 38.9] and OR 7.4 [0.8; 69.7], respectively).

**Conclusions:**

Patients with a high Pocock’s clinical score are at a high risk of death or hospitalization. For patients with a low Pocock’s clinical score, the KCCQ score can identify those at risk of these events.

**Electronic supplementary material:**

The online version of this article (doi:10.1007/s11136-015-1154-9) contains supplementary material, which is available to authorized users.

## Introduction

Heart failure (HF) is the leading cause of hospitalization in patients older than 65 years [1] with readmission rates over 40 % within the 6 months following a hospital discharge [2]. The prognosis of HF is very bad, making it a major public health problem. About half of the patients die within the 4 years following the diagnosis of HF and more than half in the year if the HF is severe [3]. Therefore, well-determining HF prognosis is important and requires an approach that cannot be limited to the use of a few biomarkers. Other health measurements such as quality-of-life indicators are useful. The Kansas City Cardiomyopathy Questionnaire (KCCQ) has been developed to quantify the health status of patients with congestive HF. It is a valid, reliable, self-administered, 23-item questionnaire that quantifies physical limitations, symptoms, self-efficacy, social interference and quality of life on a 0–100 % scale [4]. Subsequent studies have validated the KCCQ and further demonstrated that a decrease in KCCQ is associated with a poorer prognosis of hard outcome endpoints in HF patients [5, 6]. Heart failure’s prognosis in individual patients is highly variable. Assessing the prognosis of each patient based on his/her own overall risk score is useful for an appropriate management. Risk models for patients with HF exist [7, 8]. Most were developed in a single cohort of patients, and there is therefore a need to assess their generalizability. The Meta-analysis Global Group in Chronic HF (MAGGIC) includes individual data on 39,372 patients with HF [9]. In this large study, Pocock and his group established a generalizable, easy-to-use clinical score on a 0–100 % scale that increases as the risk of mortality increases [9]. The Pocock’s clinical score is comprised of observed factors and patient characteristics, while the KCCQ is self-reported by patients. These two instruments do not appear to overlap. If both of these instruments have prognostic value in HF, then one can expect a negative correlation between them, despite the fact that each questions differing aspects of the disease in patients. Using a prospective registry of HF outpatients followed by general practitioners outside of clinical settings (part of the Better Efficacy in Lowering events by General practitioner’s Intervention Using remote Monitoring in Heart Failure—BELGIUM-HF study, a remote home telemonitoring study in HF in which patients completed a six-month blind daily weight, blood pressure and pulse measurements), we assessed the relationship between the KCCQ and the Pocock’s clinical score [9], and their additive prognostic value.

## Materials and methods

### Study design

The BELGIUM-HF study was designed in 2007 and implemented in 2008–2010 in Brussels and southern Belgium. It was a prospective registry of HF outpatients followed by their general practitioners (GP) but identified from the records of 16 cardiology centers. The protocol was approved by the ethical committee of each of the 16 centers, and an informed consent form was signed by each of the participants. For each identified patient, his/her GP was contacted for the study. If the GP agreed, the patient was followed for up to 6 months by his/her GP.

### Study patients

Patients were enrolled by their general practitioners in a non-institutional environment. They were eligible if they were hospitalized for HF in the preceding 6 months, had a left ventricular ejection fraction (LVEF) <40 % and required daily loop diuretics within the preceding 2 weeks of inclusion. Excluded patients were patients <18 years of age, patients awaiting cardiac surgery or who underwent myocardial revascularization within the preceding 3 months, patients treated by or considered for chronic hemodialysis procedures and patients whose cognitive aptitudes were impaired.

### Data collection

Clinical and biological data were collected at baseline. Patients also fulfilled the KCCQ and performed the Six-Minute Walk Test.

### Health status assessment

The New York Heart Association (NYHA) classification was used to assess patients’ cardiovascular status. The NYHA class is a four-level scale assigning a functional class (from I to IV) based on physical limitations caused by cardiac symptoms. NYHA class I is defined as cardiac disease but no symptoms and no limitation in ordinary physical activity. NYHA class II describes mild symptoms and slight limitation during ordinary activity. NYHA class III is defined as an inability to perform a physical activity without symptoms. NYHA class IV describes severe limitation with symptoms even while at rest [10].

Patients enrolled in the study completed the KCCQ at baseline. The KCCQ is a 23-item, self-administered questionnaire that encompasses several domains including physical limitation, symptoms (frequency, severity and recent changes), self-efficacy, social interference and quality of life for patients with congestive HF [4]. An overall summary score is then computed by combining these individual scores. This summary score ranges from 0 to 100 %, and the higher the score, the better the quality of life [4]. The questionnaire’s validity, reliability and responsiveness to clinical change have previously been established [11, 12]. In previous studies, the overall summary score was divided into four categories that were associated with an increased risk of mortality and hospitalization for patients with decreasing scores: 0 to <24 % (*worst*); 25–49 % (*poor*); 50–74 % (*fair*); and 75–100 % (*good*) [13, 14].

### Pocock’s clinical score

The Meta-analysis Global Group in Chronic Heart Failure (MAGGIC) includes individual data on 39,372 patients with HF, both reduced and preserved left ventricular ejection fraction (EF), from 24 cohort studies and six clinical trials [9]. It established a generalizable, easy-to-use risk score of mortality in patients with HF using Poisson regression models [9]. Authors identified 13 variables as significant independent predictors of mortality in the following order: age, low LVEF, New York Heart Association (NYHA) class, serum creatinine, diabetes, absence of a prescribed beta-blocker, low systolic blood pressure, low body mass, time since diagnosis, current smoker, chronic obstructive pulmonary disease, male gender and absence of a prescribed angiotensin-converting-enzyme inhibitor or angiotensin-receptor blockers [9]. The model-derived clinical score was defined as the 0–100 rescaling of the estimated regression line, with a zero score corresponding to the lowest possible risk of a patient. Using this model, we computed a risk score of each patient of the BELGIUM-HF study.

### The Six-Minute Walk Test

The Six-Minute Walk Test is a practical simple test. It requires a 100-ft hallway but no exercise equipment or advanced training for technicians [15]. This test measures the distance that a patient can quickly walk on a flat, hard surface during a period of 6 min. “It evaluates the global and integrated responses of all the systems involved during exercise, including the pulmonary and cardiovascular systems, systemic circulation, peripheral circulation, blood neuromuscular units and muscle metabolism” [15]. Good correlations have been reported between the Six-Minute Walk Test and cardiopulmonary testing. In some clinical situations, the Six-Minute Walk Test provides information that may be a better index of the patient’s ability to perform daily activities than is peak oxygen uptake, i.e., the 6-min walking distance correlates better with measures of quality of life [16]. The test was performed according to the guidelines of the American Thoracic Society at baseline, and the total distance walked during the test was considered.

### Study outcome

The main outcome was death or hospitalization within 6 months. Both events were equally weighted to define the outcome as a dummy variable coding for any of these two events.

### Statistical analyses

Patients’ characteristics are presented as mean with standard deviation (SD) or median with quartiles for continuous variables and as number and proportion for discrete variables. Variables with a lognormal distribution are listed as geometric mean (SD). Pearson correlation was used to assess the correlation between KCCQ subscales and the NYHA class or the total walked distance at the Six-Minute Walk Test. Trends across KCCQ overall summary score categories were analyzed using Cochran–Armitage trend test for categorical variables and Spearman’s rank correlation for continuous variables. Pearson correlation was used to assess the correlation between KCCQ scores and the Pocock’s clinical score [9]. A multivariate logistic regression was performed, including the KCCQ overall summary score and the Pocock’s clinical score that were encoded into dummy variables and their interaction in the model. The significance level was set to 0.05, and all tests were two-sided. Statistical analyses were performed using SPSS 22.

## Results

**Fig. 1:**
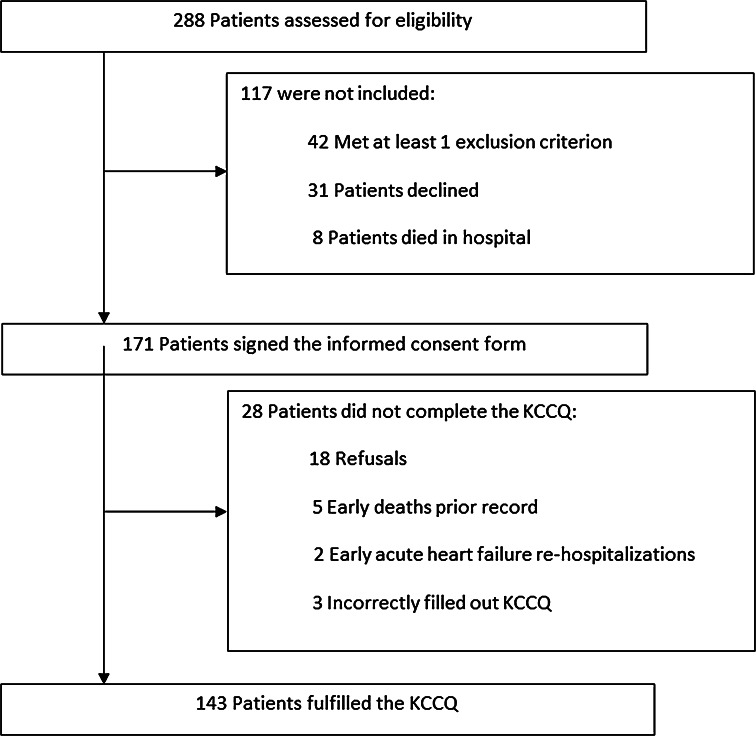
Flowchart. Out of 288 patients assessed for eligibility, 143 fulfilled the Kansas City Cardiomyopathy Questionnaire

From January 2008 to December 2010, 288 patients were eligible for study entry. However, 117 were not included because of exclusion criteria or refusal. Among the remaining 171 patients, another 28 patients did not complete the KCCQ for various reasons (Fig. 1). A total of 143 patients filled out the KCCQ at baseline. Because this study is a part of the BELGIUM-HF study, a remote home telemonitoring study in HF, patients had regular contacts with their GPs, so no hospitalization or death was lost to follow-up.

The baseline clinical characteristics of patients are reported in Table 1. Mean age of patients was 68 years, with 54 % aged >70 years, and 74 % men. New York Heart Association symptoms of class III or IV were found in 58 %.

**Table 1:** Baseline characteristics of patients

	All patients *n* = 143	Death or hospital readmission	
		Yes *n* = 61	No *n* = 82
Clinical data			
Age—years	68 ± 12	71 ± 11	66 ± 13
Male—no (%)	106 (74.1)	46 (75.4)	62 (75.6)
Body mass index (kg/m^2^)	27.0 ± 4.8	26.9 ± 3.7	27.0 ± 5.5
Systolic blood pressure (mmHg)	113 ± 26	112 ± 20	114 ± 29
Diastolic blood pressure (mmHg)	69 ± 17	67 ± 14	70 ± 19
NYHA class III–IV—no (%)	83 (58.0)	41 (67.2)	43 (52.4)
Risk factors			
Hypercholesterolemia—no (%)	74 (51.7)	34 (55.7)	40 (48.8)
Hypertension—no (%)	82 (57.3)	31 (50.8)	51 (62.2)
Diabetes—no (%)	47 (32.9)	22 (36.1)	25 (30.5)
Smoker within past 12 months—no (%)	37 (25.9)	12 (19.7)	25 (30.5)
Six-Minute Walk Test—median (IQR)	290 (200–380)	219 (160–311)	300 (238–420)
Biological data^a^			
Brain natriuretic peptide (pg/ml)	741.3 (3.2)	853.9 (3.2)	621.0 (3.2)
Glucose (mg/dl)	107.2 (1.3)	109.2 (1.4)	105.7 (1.3)
Cholesterol (mg/dl)	147.9 (1.3)	137.1 (1.3)	156.9 (1.4)
LDL cholesterol (mg/dl)	79.4 (1.5)	70.5 (1.5)	84.0 (1.6)
Creatinine (mg/dl)	1.44 (1.45)	1.48 (1.48)	1.34 (1.35)
Left ventricular ejection fraction (%)	28 ± 7	28 ± 8	28 ± 7
Renal failure—no (%)	11 (7.7)	7 (11.5)	4 (4.9)
Chronic obstructive pulmonary disease—no (%)	22 (15.4)	11 (18.0)	11 (13.4)

Plus minus data are mean ± SD

*NYHA* New York Heart Association, *LDL* low-density lipoprotein

^a^Data are geometric means (SD)

**Fig. 2:**
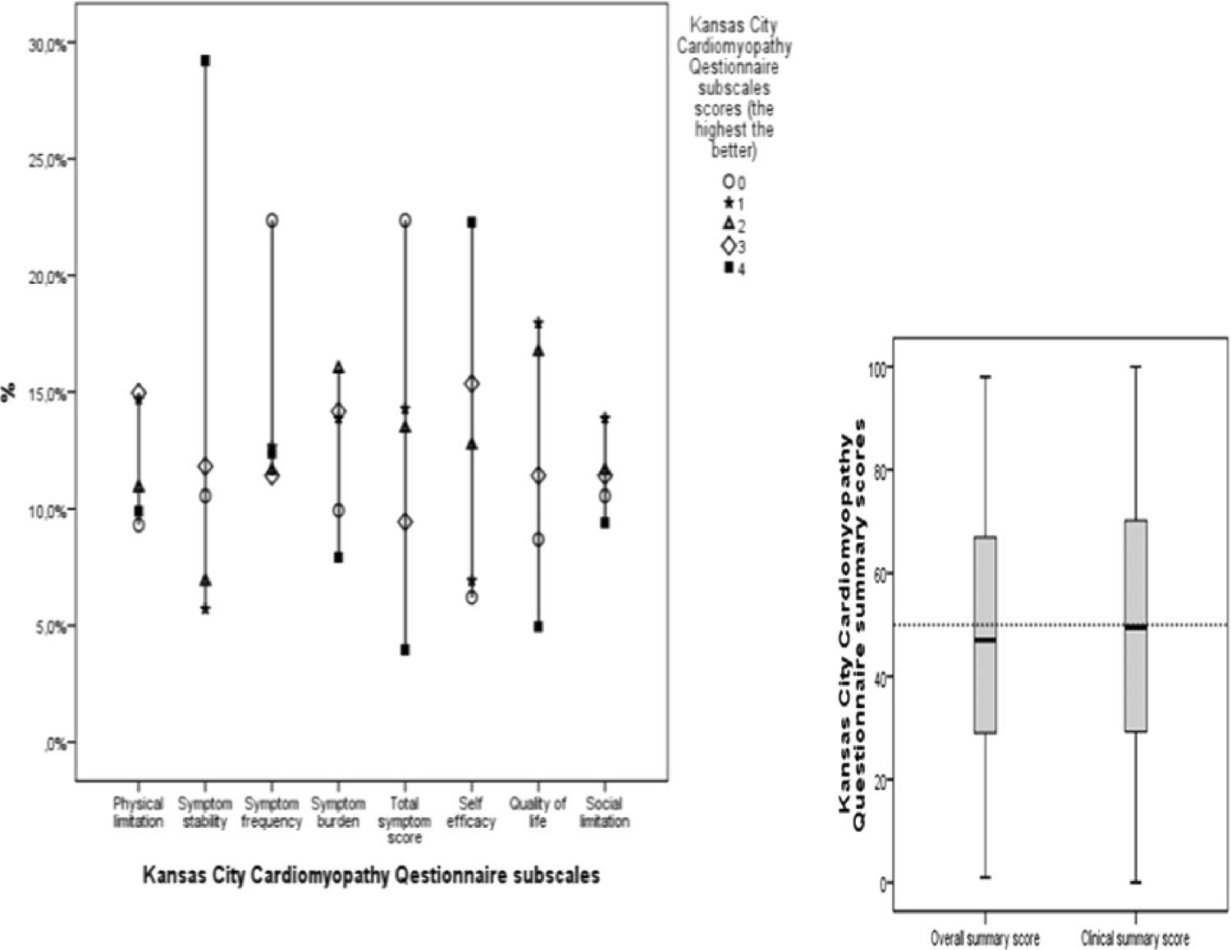
Distribution of Kansas City Cardiomyopathy Questionnaire subscales and summary scores in patients with heart failure. At baseline, most of the patients had symptoms remission and felt self-efficacy. Nearly half of the patients had an overall summary score of at least 50 %

Figure 2 shows the distribution of the KCCQ’s subscales and summary scores. At baseline, patients’ symptoms were stabilized and they had a positive perception of self-efficacy . Nearly half of them had a KCCQ’s overall summary score of at least 50 at baseline.

The difference between the cumulative KCCQ’s overall summary score distribution of patients who experienced an HF outcome and those who did not experience an event was not Gaussian along the continuous level of the KCCQ score. Indeed, the two curves were not sigmoids, and the difference was larger for KCCQ scores around 25 %. Consequently, the KCCQ score could not be considered as a continuous scale, and it was more relevant to considering a four-point scale (quartiles) than a hundred-point scale (Online Resource 1).

Table 2 summarizes the 13 Pocock’s independent predictors of mortality in patients with HF and their relationship with the previously described categories of the KCCQ’s overall summary score [9]. Only 18.9 % of patients reported a good KCCQ’s overall summary score at baseline (≥75 %). A worst KCCQ’s overall summary score (≤25 %) was found in 22.4 % of patients. The proportion of patients with the most severe symptoms on NYHA classification increased as the KCCQ’s overall summary score decreased (trend *p* < 0.001). The same observation was made for the proportion of patients with a chronic obstructive pulmonary disease (trend *p* = 0.008). Such variables are measures of physical capacities that are expected to be correlated with tools measuring perceived health such as the KCCQ. In contrast, other variables such as creatininemia and the use or not of beta-blockers are not expected to reflect a perceived health. When taking into account all of the 13 variables to compute Pocock’s clinical score, it was observed that the mean Pocock’s clinical score increased as the KCCQ’s overall summary score decreased (trend *p* = 0.04). KCCQ’s overall summary score was inversely correlated with the Pocock’s clinical risk score (*r* = −0.24, *p* = 0.026).

**Table 2:** Baseline variables identified by Pocock with trends across the Kansas City Cardiomyopathy Questionnaire’s (KCCQ)

	All patients *n* = 143	KCCQ overall summary score categories				
		Worst (<25) *n* = 32	Poor (25–49) *n* = 45	Fair (50–74) *n* = 39	Good (75–100) *n* = 27	Trend test *p* value
Age—years	68 ± 12	67 ± 13	71 ± 12	67 ± 13	67 ± 11	0.57
Left ventricular ejection fraction (%)	28 ± 7	28 ± 7	27 ± 6	27 ± 8	29 ± 8	0.40
NYHA class III–IV—no (%)	83 (58.0)	24 (75.0)	35 (77.8)	18 (46.2)	6 (22.2)	<0.001
Creatinine^a^ (mg/dl)	1.44 (1.45)	1.30 (1.44)	1.50 (1.4)	1.45 (1.44)	1.30 (1.44)	0.49
Diabetes—no (%)	46 (32.2)	11 (34.4)	9 (20.0)	17 (43.6)	9 (33.3)	0.45
Beta-blocker—no (%)	114 (79.7)	30 (93.8)	34 (75.6)	29 (74.4)	21 (77.8)	0.13
Systolic blood pressure (mmHg)	113 ± 26	116 ± 20	115 ± 23	110 ± 32	107 ± 27	0.12
Body mass index (kg/m^2^)	27.0 ± 4.8	28.4 ± 5.9	26.2 ± 5.4	26.7 ± 4.3	27.0 ± 3.4	0.92
Time since diagnosis (months)	38 ± 44	41 ± 45	41 ± 37	44 ± 55	23 ± 35	0.09
Smoker within past 12 months—no (%)	36 (25.2)	11 (34.4)	9 (20.0)	7 (17.9)	9 (33.3)	0.80
COPD—no (%)	22 (15.4)	9 (28.1)	8 (17.8)	4 (10.3)	1 (3.7)	0.008
Male—no (%)	106 (74.1)	23 (71.9)	32 (71.1)	28 (71.8)	23 (85.2)	0.29
ACEor Sartan—no (%)	135 (94.4)	27 (84.4)	44 (97.8)	38 (97.4)	26 (96.3)	0.07
Pocock’s clinical score	34 ± 15	38 ± 16	36 ± 14	34 ± 16	31 ± 14	0.04

Plus minus data are mean ± SD

*COPD* chronic obstructive pulmonary disease, *ACE* angiotensin-converting-enzyme inhibitor

^a^Data are geometric means (SD)

Other patient characteristics such as the proportion of patients in sinus rhythm (trend *p* = 0.04) and the mean walk distance at the Six-Minute Walk Test (trend *p* = 0.04) decreased with the KCCQ’s overall summary score (Table 3). Mean diastolic blood pressure (trend *p* = 0.32), mean pulse rate (trend *p* = 0.09), the proportion of patients with pulmonary rhoncus (trend *p* < 0.001) and those with peripheral edema (trend *p* = 0.003) increased as the KCCQ’s overall summary score decreased (Table 3). We also observed that brain natriuretic peptide levels were the highest in patients with a low KCCQ’s overall summary score (Table 3).

**Table 3:** Patients’ other characteristics

	All patients *n* = 143	KCCQ overall summary score categories				
		Worst (<25) *n* = 32	Poor (25–49) *n* = 45	Fair (50–74) *n* = 39	Good (75–100) *n* = 27	Trend test *p* value
Clinical data						
Diastolic blood pressure (mmHg)	69 ± 17	72 ± 14	68 ± 14	68 ± 21	67 ± 18	0.32
Pulse rate—beats per minute	78 ± 18	81 ± 16	78 ± 16	78 ± 21	76 ± 19	0.09
Sinusal rhythm—no (%)	109 (76.2)	23 (71.9)	35 (77.8)	29 (74.4)	22 (81.5)	0.04
Pulmonary rhoncus—no (%)	47 (32.9)	14 (43.8)	18 (40.0)	8 (20.5)	7 (25.9)	<0.001
Jugular distension—no (%)	36 (25.2)	13 (40.6)	16 (35.6)	6 (15.4)	1 (3.7)	0.003
Peripheral edema—no (%)	52 (36.4)	15 (46.9)	24 (53.3)	7 (17.9)	6 (22.2)	
Six-Minute Walk Test distance (m)	298 ± 130	258 ± 138	280 ± 149	311 ± 127	334 ± 105	0.04
Biological data^a^						
Brain natriuretic peptide (pg/ml)	741.3 (3.2)	977.2 (2.9)	630.9 (3.6)	660.7 (2.6)	676.1 (3.4)	0.29
Glucose (mg/dl)	107.2 (1.3)	107.2 (1.3)	100.0 (1.2)	109.6 (1.4)	114.8 (1.5)	0.51
Troponin (µg/l)	0.034 (2.6)	0.032(0.347)	0.030 (2.820)	0.033 (1.995)	0.047 (2.754)	0.47

Plus minus data are mean ± SD

^a^Data are geometric means (SD)

When looking at the correlation between each of the KCCQ subscales and the NYHA class, we found all significant negative correlations except for the self-efficacy subscale (*p* = 0.09), showing low KCCQ values in patients from high NYHA classes. In addition, the walk distance for the Six-Minute Walk Test was positively correlated with all KCCQ subscales except the self-efficacy subscale (*p* = 0.16), similarly showing low walk distances in patients with lower KCCQ values, who are also the frailest patients (Table 4).

**Table 4:** Correlations between KCCQ subscales and NYHA class or Six-Minute Walk Test

	NYHA	*p* value	Walk distance	*p* value
Kansas City Cardiomyopathy Questionnaire subscales				
Physical limitation	−0.46	<0.001	0.18	0.04
Symptom stability	−0.08	0.02	0.33	0.01
Symptom frequency	−0.43	<0.001	0.31	0.01
Symptom burden	−0.40	<0.001	0.26	0.04
Total symptom score	−0.43	<0.001	0.30	0.02
Self-efficacy	−0.14	0.09	0.18	0.16
Quality of life	−0.33	<0.001	0.27	0.03
Social limitation	−0.39	<0.001	0.24	0.07
Overall summary score	−0.44	<0.001	0.27	0.03
Clinical summary score	−0.46	<0.001	0.25	0.04

During the 6 months of follow-up, 10 (7.0 %) patients died. In addition, 51 (35.7 %) were reported hospitalized, resulting in 42.7 % of patients presenting an event during the 6 months following study start. Patients who experienced an event were older and had more severe symptoms on NYHA classification (Table 1). They also had higher levels of brain natriuretic peptide and a smaller mean walk distance at the Six-Minute Walk Test (Table 1).

**Fig. 3:**
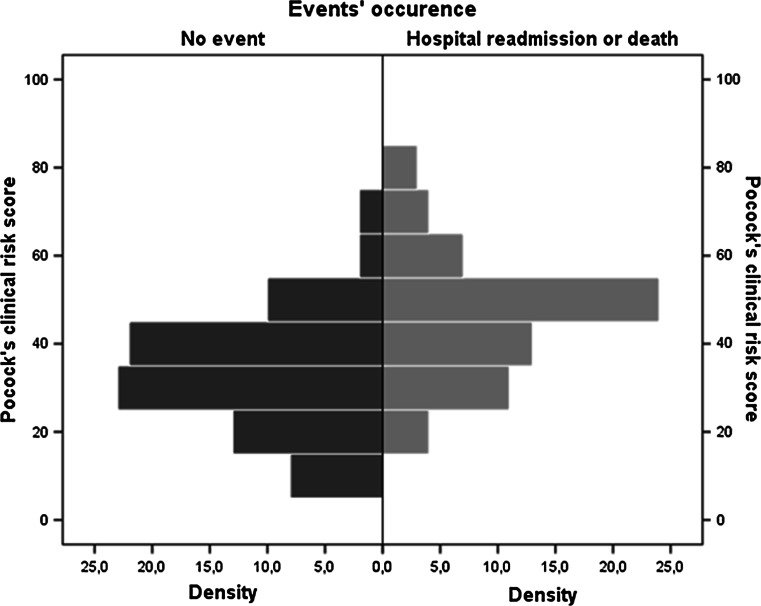
Histogram of Pocock’s clinical score according to event occurrence. Patients who experienced an event had the highest Pocock’s clinical scores at baseline

Patients who experienced an event had a higher Pocock’s clinical score at baseline as illustrated in Fig. 3.

**Fig. 4:**
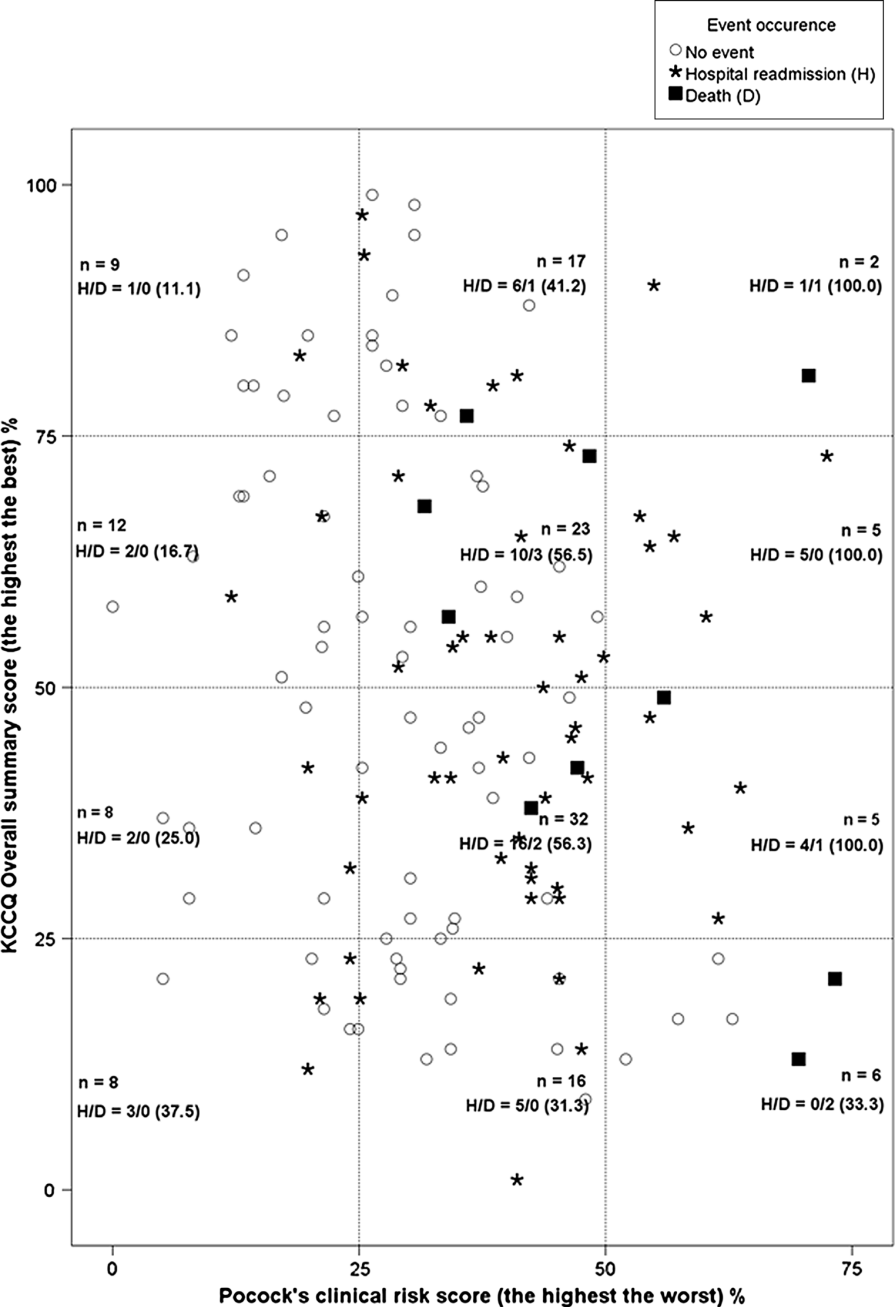
Kansas City Cardiomyopathy Questionnaire’s overall summary score and Pocock’s clinical score according to event occurrence. Patients with a significantly affected Pocock’s clinical score (>50 %) have almost all experienced an event. For patients with a slightly affected Pocock’s clinical sore (≤50 %) at baseline, we observed a morbi-mortality gradient according to the KCCQ overall summary score. Indeed, the proportion of events in these patients increased as their KCCQ overall summary score worsened

Almost all patients with a high Pocock’s clinical score (≥50 %) experienced an event regardless of the KCCQ’s overall summary score. For patients with a medium (25–50 %) or a low (≤25 %) Pocock’s clinical score, we observed a morbi-mortality gradient according to the KCCQ’s overall summary score. Indeed, the number of events observed in these patients increased as their KCCQ’s overall summary score worsened (Fig. 4).

Using multivariate logistic regression, the estimated equation for the KCCQ overall summary score and the Pocock’s clinical score was the following:

$$\begin{aligned} {\text{Logit}}(p) & = - 2.303 + 1.996 \times {\text{Pocock}}_{25 - 50\,\% } + 1.514 \\ & \quad \times {\text{KCCQ}}_{ \le 50\,\% } + 0.693 \times {\text{KCCQ}}_{50 - 75\,\% } \\ & \quad - 1.304 \times {\text{Pocock}}_{25 - 50\,\% } \times {\text{KCCQ}}_{ \le 50\,\% } \\ & \quad - 0.059 \times {\text{Pocock}}_{25 - 50\% } \times {\text{KCCQ}}_{50 - 75\,\% } \\ \end{aligned}$$

A significant interaction was found between the KCCQ overall summary score and the Pocock’s clinical score. This interaction is illustrated in Table 5. There was a high proportion of events (14/18 = 77.8 %) in patients with a high (>50 %) Pocock’s clinical score, whatever the KCCQ score value. When the KCCQ score was ≤50 % (top panel), there was a low increase in risk as the Pocock’s clinical score increased (OR 2.00, 95 % CI 0.60–6.62). But when the KCCQ score was between 50 and 75 % (middle panel) or ≥75 % (lower panel), there was a high increase in risk as the Pocock’s clinical score increased (OR 6.94, 95 % CI 1.24–38.86, and OR 7.36, 95 % CI 0.78–69.70, respectively).

**Table 5:** Multivariate logistic regression: predictive value of Pocock’s clinical score and Kansas City Cardiomyopathy Questionnaire’s overall summary score

	*n*	Events *n* (%)	OR	IC_95 %_^a^ (OR)
KCCQ overall summary score: high risk (≤50 %)				
Pocock’s clinical score: high risk (≥50 %)	11	7 (63.6)	Not computed^b^	
Pocock’s clinical score: medium risk (25–50 %)	48	23 (47.9)	2.00	[0.60; 6.62]
Pocock’s clinical score: low risk (≤25 %)	16	5 (31.3)	1	
KCCQ overall summary score: medium risk (50–75 %)				
Pocock’s clinical score: high risk (≥50 %)	5	5 (100.0)	Not computed^b^	
Pocock’s clinical score: medium risk (25–50 %)	23	13 (56.5)	6.94	[1.24; 38.86]
Pocock’s clinical score: low risk (≤25 %)	12	2 (16.7)	1	
KCCQ overall summary score: low risk (≥75 %)				
Pocock’s clinical score: high risk (≥50 %)	2	2 (100.0)	Not computed^b^	
Pocock’s clinical score: medium risk (25–50 %)	17	7 (41.2)	7.36	[0.78; 69.70]
Pocock’s clinical score: low risk (≤25 %)	9	1 (11.1)	1	

*OR* odds ratio

^a^95 % confidence interval

^b^Because of 64–100 % events

Comparing the KCCQ subscales mean scores with respect to the occurrence of death or hospital admission in the subgroup of patients with a medium or a low Pocock’s clinical score, we observed that patients who experienced an event had lower mean scores for each of these ten subscales. The difference between mean scores was about 3 for all subscales except for self-efficacy, where it reached 10 (Online Resource 2).

## Discussion

The purpose of this study was to assess the relationship and the additive prognostic value of the KCCQ, a validated measurement tool for patient-perceived health status in congestive HF, and the Pocock’s clinical score of mortality in HF patients. We found an inverse correlation between the KCCQ’s overall summary score and the Pocock’s clinical score. This indicates that patients with a significantly affected Pocock’s clinical score also tend to have a poorer quality of life. When Pocock’s clinical score is high (>50 %), there is no additional prognostic value of KCCQ’s overall summary score for hospital admission or death within 6 months. However, when Pocock’s clinical score is medium (25–50 %) or low (≤25 %), KCCQ’s overall summary score has a significant prognostic value. Therefore, these two instruments are complementary ways to assess the progression of HF.

Pocock’s clinical score has an interesting prognostic value in that patients with a high Pocock’s clinical score almost all experienced an event.

As already established [4], we reported a negative correlation between KCCQ and NYHA class and a positive correlation between KCCQ and walk distance at the Six-Minute Walk Test. Nonetheless, we observed that the KCCQ’s self-efficacy subscale was not significantly correlated with the NYHA class or to the walk distance at the Six-Minute Walk Test, in contrast to the other nine subscales of the KCCQ. This reflects the fact that most KCCQ subscales and the NYHA classification assess the patient’s physical limitations. For the KCCQ subscales, this limitation is based on the patient’s perspective, and for the NYHA classification, it is based on the physician’s perspective [17, 18]. In contrast, the self-efficacy subscale assesses a very subjective aspect related to the knowledge of the patient about his/her HF. This is consistent with the findings of a recent study conducted to reconceptualize KCCQ subscales and to advance its use, more than 10 years after its publication [19].

We further observed that patients who experienced an event had the lowest mean scores for all KCCQ’s subscales. The difference between these mean scores was the highest for the self-efficacy subscale. Thus, the KCCQ’s self-efficacy subscale does not seem consistent with the other KCCQ’s subscales, but it evaluates an important health status, i.e., patient autonomy, and requires thorough assessment.

Our study has limitations, and the small sample size is the main one. Nevertheless, despite our small number of patients, there are several arguments proving the quality of our data base. Firstly, as we have just discussed, patients were recruited by their general practitioners in a non-institutional environment. Such a sample is smaller, but it is more representative of patients with HF in their daily life, outside of hospitals. In this respect, it is original and deserves special attention. Secondly, with a small sample, we observed 42.7 % of events at 6 months, which is consistent with the 40 % [2] reported in the literature. Thirdly, we found consistent correlations between all the KCCQ subscales and the NYHA class and walk distance at Six-Minute Walk test, as already established. Finally, we found the prognostic value of the Pocock’s clinical score. All these give a power to generalize our findings.

In conclusion, patients with a high Pocock’s clinical score are at a high risk of death or hospitalization during a 6-month follow-up and the KCCQ does not add prognostic value. For patients with a low Pocock’s clinical score, the KCCQ score allows for further characterization of a still high risk group. That information might lead to targeting therapies interventions to mitigate that group’s risk of death or hospitalization. These two instruments are therefore complementary in assessing health in its globality for HF patients. KCCQ may therefore represent a different approach in HF management.

## Electronic supplementary material

Below is the link to the electronic supplementary material.

Supplementary material 1 (DOCX 67 kb)

Supplementary material 2 (DOCX 18 kb)
