# Supplementary material for: Interaction between the Kansas City Cardiomyopathy Questionnaire and the Pocock’s clinical score in predicting heart failure outcomes
Source: Qual Life Res. 2015 Oct 11;25:1245–55. doi: 10.1007/s11136-015-1154-9 (PMC4840225; doi:10.1007/s11136-015-1154-9)
Supplement: Supplementary file 1 — Supplementary material 1 (DOCX 67 kb) [file 11136_2015_1154_MOESM1_ESM.docx]

Mean ± SD

: 43 ± 25

: 51 ± 22


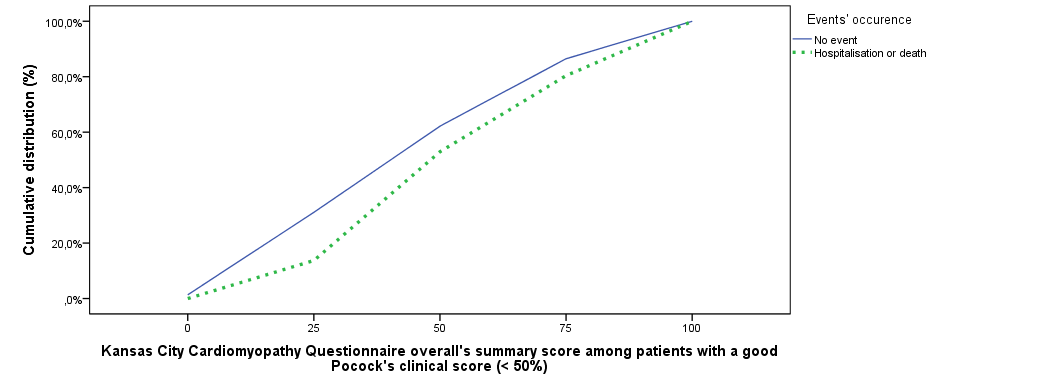


**Online Resource 1. Cumulative distribution of the KCCQ score according to HF outcome**. Patients who experienced an HF outcome had lower cumulative KCCQ scores than those who did not. But the difference between the two curves was neither a sigmoid nor a maximum at the mean difference of the KCCQ
