# Supplementary material for: Interaction between the Kansas City Cardiomyopathy Questionnaire and the Pocock’s clinical score in predicting heart failure outcomes
Source: Qual Life Res. 2015 Oct 11;25:1245–55. doi: 10.1007/s11136-015-1154-9 (PMC4840225; doi:10.1007/s11136-015-1154-9)
Supplement: Supplementary file 2 — Supplementary material 2 (DOCX 18 kb) [file 11136_2015_1154_MOESM2_ESM.docx]

| Online Resource 2. Comparison of KCCQ subscale means according to events’ occurrence in the subgroup of patients with a medium (25-50%) or low (≤ 25%) Pocock’s clinical score | | | | |
| --- | --- | --- | --- | --- |
|  |  | | **Death or hospital readmission** | |
|  | | | **Yes (n = 61)** | **No (n = 82)** |
|  | **Physical limitation** | | | |
|  |  | Mean | 51 ± 29 | 53 ± 31 |
|  |  | Median (IQR) | 50 (25-75) | 50 (25-75) |
|  | **Symptom stability** | | | |
|  |  | Mean | 67 ± 35 | 70 ± 35 |
|  |  | Median (IQR) | 75 (44-100) | 75 (50-100) |
|  | **Symptom frequency** | | | |
|  |  | Mean | 51 ± 30 | 54 ± 27 |
|  |  | Median (IQR) | 21 (50-79) | 56 (33-76) |
|  | **Symptom burden** | | | |
|  |  | Mean | 48 ± 24 | 50 ± 29 |
|  |  | Median (IQR) | 50 (25-75) | 50 (25-75) |
|  | **Total symptom score** | | | |
|  |  | Mean | 48 ± 24 | 52 ± 26 |
|  |  | Median (IQR) | 47 (26-73) | 49 (32-69) |
|  | **Self-efficacy** | | | |
|  |  | Mean | 56 ± 28 | 66 ± 29 |
|  |  | Median (IQR) | 50 (38-75) | 75 (50-88) |
|  | **Quality of life** | | | |
|  |  | Mean | 46 ± 23 | 49 ± 26 |
|  |  | Median (IQR) | 50 (33 -58) | 50 (25-67) |
|  | **Social limitation** | | | |
|  |  | Mean | 47 ± 31 | 52 ± 31 |
|  |  | Median (IQR) | 50 (25-73) | 50 (25-77) |
|  | **Overall summary score** | | | |
|  |  | Mean | 48 ± 23 | 51 ± 25 |
|  |  | Median (IQR) | 43 (31-68) | 53 (29-71) |
|  | **Clinical summary score** | | | |
|  |  | Mean | 48 ± 24 | 52 ± 26 |
|  |  | Median (IQR) | 41 (30-70) | 53 (29-72) |
